# One-year outcomes of fixed-dosing Aflibercept therapy for pre treated and naive polypoidal choroidal vasculopathy patient

**DOI:** 10.1186/s12886-021-01829-2

**Published:** 2021-02-18

**Authors:** Hun Gu Choo, Jin Hae Lee, Hyun Sub Oh, Soon Hyun Kim, Yong Sung You, Oh Woong Kwon

**Affiliations:** 1 Department of Ophthalmology, Wonju College of Medicine – Yonsei University, Wonju, Republic of Korea; 2 Department of Ophthalmology, First St. Marys Eye Clinic, Seoul, Republic of Korea; 3 Department of Ophthalmology, Nune Eye Hospital, Noon Bldg 404, Seonreung-ro Gangnam-gu, 06198 Seoul, Republic of Korea

**Keywords:** Polypoidal choroidal vasculopathy, Age-related macular degeneration, Aflibercept, Fixed-dosing regimen

## Abstract

**Background:**

Polypoidal choroidal vasculopathy (PCV) is a type of age-related macular degeneration that can cause permanent vision loss. The purpose of this paper was to report the one-year outcomes of fixed-dosing aflibercept therapy for the treatment of PCV.

**Methods:**

This was a prospective, single-arm, interventional case series study of 25 PCV patients; 12 pre-treated and 13 treatment-naïve patients. The patients were treated and monitored for 12 months. Each patient was administered with an aflibercept (2.0 mg) injection every month for the first 3 months (the loading phase), and thereafter, once every 2 months. At every follow-up visit, best-corrected visual acuity (BCVA) test, fundus examination, and optical coherence tomography for measuring the central subfield macular thickness (CSMT) were performed. Fluorescein and indocyanine green angiography were conducted at baseline and at 4 and 12 months.

**Results:**

After 12 months of aflibercept therapy, the mean BCVA of the patients significantly improved from 65.48 letters at baseline to 69.91 letters (*p*=0.001), and the CSMT significantly decreased from 406.92 um at baseline to 276.12 um (*p*< 0.001). Additionally, ten patients (40%) showed complete polyp regression. The treatment-naïve patients showed a statistically significant improvement in BCVA from 66.58 letters at baseline to 76.36 letters at 12 months, and a significant decrease in CSMT, from 462 to 243 um. In the pre-treated group, there was no change in BCVA (64.46 letters), and the decrease in CSMT from 356.08 to 303.69 um was not statistically significant.

**Conclusions:**

The fixed-dosing aflibercept regimen is effective for treating patients with PCV and is more effective in treatment-naïve patients than in pre-treated patients.

**Trial registration:**

Clinical Research Information Service (CRiS), Republic of Korea. Identifer: KCT0005798, Registered: Jan 20, 2021. Retrospectively registered, URL: https://cris.nih.go.kr/cris/en/search/search_result_st01.jsp?seq=18546

## Background

Polypoidal choroidal vasculopathy (PCV) is a subtype of age-related macular degeneration (AMD). It is characterized by hemorrhage, serous exudates, and scar formation, all of which can lead to permanent vision loss [1]. The incidence of exudative AMD is particularly high among the Asian population. In the West, only 4–9.8% of all exudative AMD cases are reported to be PCV [2–4], whereas in the East, 50% of exudative AMD cases are PCV cases [5]. This difference is attributed to underdiagnosis due to lesser utilization of indocyanine green angiography (ICGA) examination modalities, and due to differences in the racial characteristics of the populations.

Limited studies have been conducted on the treatment regimen and natural progression of PCV compared with classic AMD [6]. Studies on anti-vascular endothelial growth factor (VEGF) therapy have established it as a first-line therapy for AMD [6, 7]. However, whether the treatment should be used as a first-line therapy for treating PCV remains controversial [8]. Photodynamic therapy (PDT) is one of the therapeutic methods that have been used for treating PCV. A study reported that PDT is more effective than anti-VEGF therapy as a short-term therapy for improving the anatomical signs of PCV, such as persistent subretinal fluid or polypoidal vessels [9], despite anti-VEGF being superior in terms of the functional outcomes of vision [10]. The EVEREST study, in which ranibizumab (an anti-VEGF agent) monotherapy was compared with PDT combined therapy, showed that PDT combined therapy resulted in a higher polyp regression rate and a higher subretinal fluid resolution rate compared with ranibizumab monotherapy without PDT, and thus, should be recommended as a first-line treatment [11]. Since the release of aflibercept, however, many studies have reported that aflibercept monotherapy yielded favorable functional outcomes and a favorable polyp closure rate when used to treat PCV [12, 13].

Reported herein are the anatomical and functional outcomes of treating PCV patients with fixed dosing aflibercept therapy for 1 year. The data provided in this paper will be helpful in establishing a new PCV treatment protocol.

## Methods

This was a prospective, single-arm, single-center, interventional case series study conducted with the support of Bayer Korea, the manufacturer of aflibercept. This study was approved by the Nune Eye Hospital Institutional Review Board/Ethics Committee((IRB no: N-1312-001-023) and it complied with the tenets of the Declaration of Helsinki. All participants provided written informed consent.

**Fig. 1:**
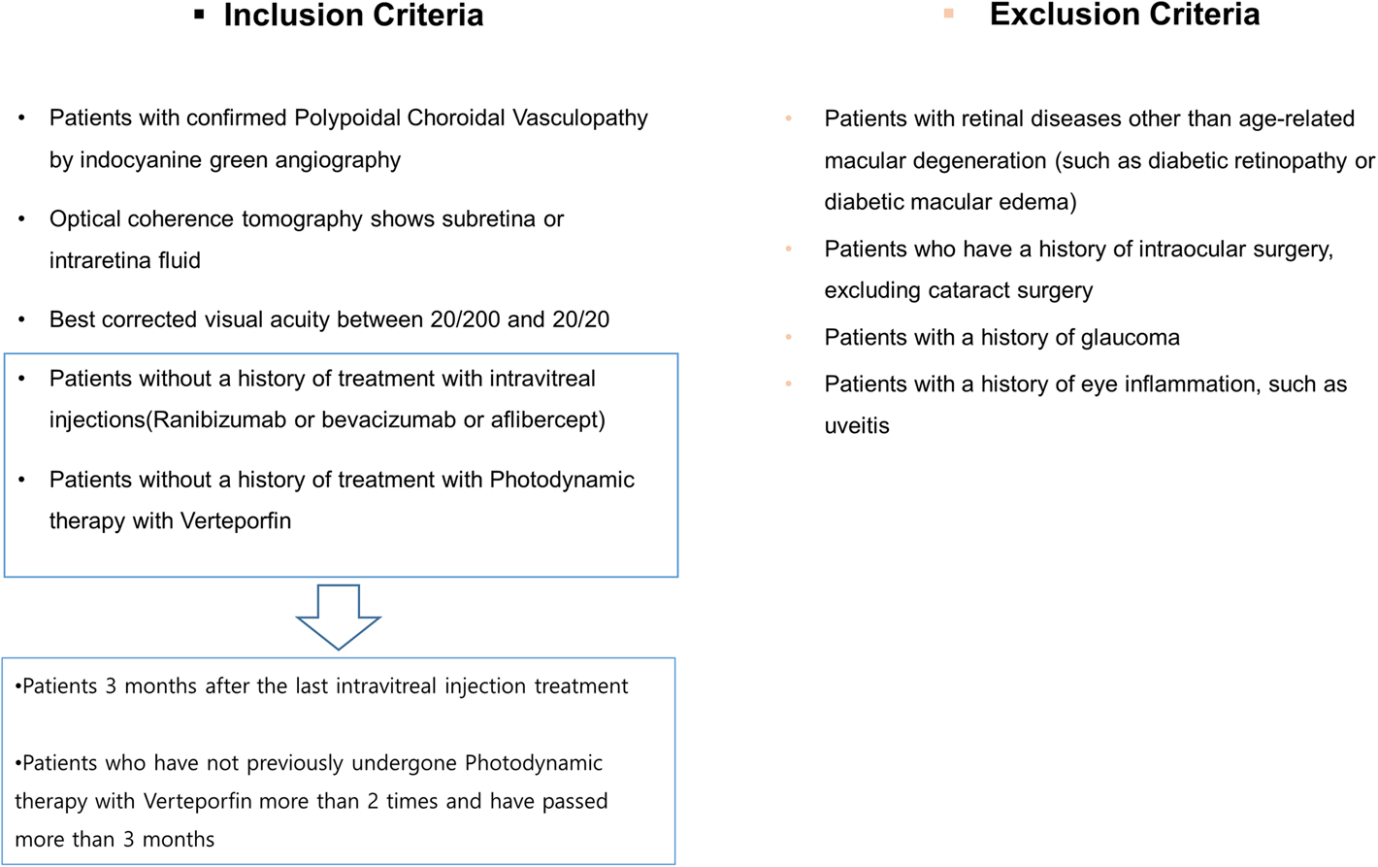
The inclusion and exclusion criteria

The study was conducted from June 2014 to March 2017. Initially, only treatment-naïve patients diagnosed with PCV for the first time were targeted. However, the inclusion criteria were revised to include pre-treated patients due to the limited number of treatment-naïve patients who could be enrolled in the study. Thirty patients were then included in the study and 25 patients completed a one-year follow-up. The inclusion and exclusion criteria are shown in Fig. 1.

Each patient received aflibercept (2.0 mg) injections (fixed dosing) in an aseptic operation room. For the loading phase, each patient was administered with an aflibercept injection once a month for 3 months; subsequently, maintenance injections were administered every 2 months. For 12 months, seven injections were administered at baseline and at the one-, two-, four-, six-, eight-, and ten-month follow-up visits. The treatment outcomes were finally evaluated at 12 months.

At every follow-up visit after the initial baseline visit, best-corrected visual acuity (BCVA) was measured using the Early Treatment Diabetic Retinopathy Study (ETDRS) chart; intraocular pressure measurement, slit lamp examination, fundus photography, and optical coherence tomography (Spectralis, Heidelberg Engineering, Heidelberg, Germany) were performed as well. At baseline and at four and 12 months, fluorescein and indocyanine green angiography (ICGA) (HRA system; Heidelberg Engineering, Heidelberg, Germany) were performed.

The primary outcome measures assessed include the following: mean change in BCVA from baseline to 12 months, proportion of patients who had complete intraretinal/subretinal fluid resolution after 12 months, and proportion of patients who had complete polypoidal lesion disappearance after 12 months based on ICGA results.

In evaluating polyp regression, complete disappearance of polyp was defined as complete regression, the decrease in the number of polyps was defined as partial regression, and no change in the number of polyps was defined as persistent polyp.

One researcher interpreted all the study images. For statistical analysis, the paired t-test, t-test, chi-squared test, and linear-by-linear association test were performed using the SPSS statistical software (ver. 24.0 for Windows, IBM Corp., Armonk, NY, USA). Statistical significance was set at *p* < 0.05.

## Results

Altogether, 25 patients were enrolled in this study: 13 pre-treated patients and 12 treatment-naïve patients. For the 12 patients in the pre-treated group, the mean disease duration was 50.53±32.17 (5–96) months and the mean frequency of anti-VEGF injections was 18.23±15.41 (3–45) times; seven patients had a history of PDT, six of whom received PDT once and one, twice.

The mean age of all the patients was 67.16±8.20 years; 20 patients were male and five were female. Fifteen right eyes and ten left eyes were included. The mean visual acuity measured at baseline was 65.48±15.43 and the baseline central macular thickness was 406.92±159.36 um; the mean number of polyps observed at baseline was 2.6. There were significant differences between the branching vascular network areas, the number of polyps, and the location of the polyps of the patients in the pre-treated and treatment-naïve groups. For the pre-treated patients, a significantly larger branching vascular network area (3.66±2.18 vs. 2.11±1.02, *p*-value=0.045) and a higher number of polyps were observed (3.30±1.88 vs. 1.83±1.02, *p*-value=0.025). For the patients in the pre-treated group, most of their lesions were located outside the fovea (extrafoveal), whereas for the patients in the treatment-naïve group, most of the lesions were located underneath the fovea (subfoveal) (*p*-value=0.004) (Table 1).

**Table 1:** Baseline characteristics of patients with polypoidal choroidal vasculopathy who were treated with a fixed-dosing intravitreal aflibercept regimen

	All	Pre-treated	Treatment-naïve	*P* value Pre-treated vs. Treatment-naïve
Numbers (eyes)	25	13	12	
Age (years)	67.16±8.20	67.2±5.44	67.12±10.7	0.983 ^a^
Sex (male: female)	20: 5	9:4	11:1	0.161^b^
Laterality (right: left)	15: 10	7:6	8:4	0.513^b^
Best Corrected Visual acuity				
Snellen	0.53±0.31	0.52±0.33	0.54±0.30	0.841 ^a^
ETDRS letters	65.48±15.43	64.46±16.52	66.58±14.79	0.739 ^a^
Central macular thickness (μm)	406.92±159.36	356.08±145.58	462±160.99	0.097 ^a^
Location of vascular lesion (eyes)				
Subfoveal	15 (60.0%)	7 (53.8%)	8 (66.6%)	0.566^c^
Juxtafoveal	9 (36.0%)	5 (38.5%)	4 (33.3%)	
Extrafoveal	1 (4.0%)	1 (8%)	0 (0%)	
Area of branching vascular network (mm^2^)	3.12±1.98	3.66±2.18	2.11±1.02	0.045^a^
Numbers of polypoidal lesions	2.60±1.68	3.30±1.88	1.83±1.02	0.025^a^
Height of the largest polypoidal lesion (μm)	345.64±159.44	331.84±134.21	360.58±187.99	0.662 ^a^
Location of the polyp closest to the foveal center (eyes)				
Subfoveal	11 (44.0%)	3 (23.0%)	8 (66.6%)	0.004^c^
Juxtafoveal	7 (28.0%)	3 (23.0%)	4 (33.3%)	
Extrafoveal	7 (28.0%)	7 (53.8%)	0 (0%)	

*ETDRS* Early Treatment of Diabetic Retinopathy Study, ^a^ T-test ^b^ Chi-squared test) ^c^ linear by linear association test

**Fig. 2:**
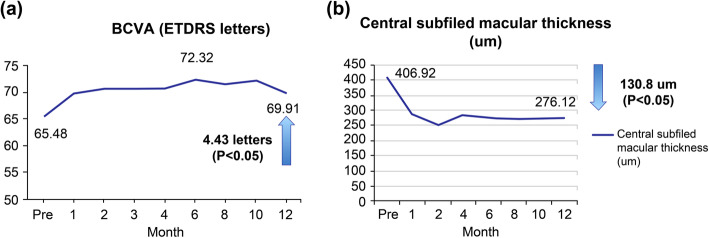
**a** Mean change in best-corrected visual acuity and **b** central subfield macular thickness

After 12 months of aflibercept treatment, the mean BCVA of the patients statistically significantly improved from 65.48 letters at baseline to 69.91 letters; CSMT significantly decreased from 406.92 um at baseline to 276.12 um at 12 months. Rapid improvement in BCVA was noted as early as 1 month after the first injection and was maintained throughout the 12 months of follow-up. Central macular thickness significantly decreased after the first two intravitreal aflibercept injections and was maintained throughout the 12 months of follow-up as well (Fig. 2).

**Fig. 3:**
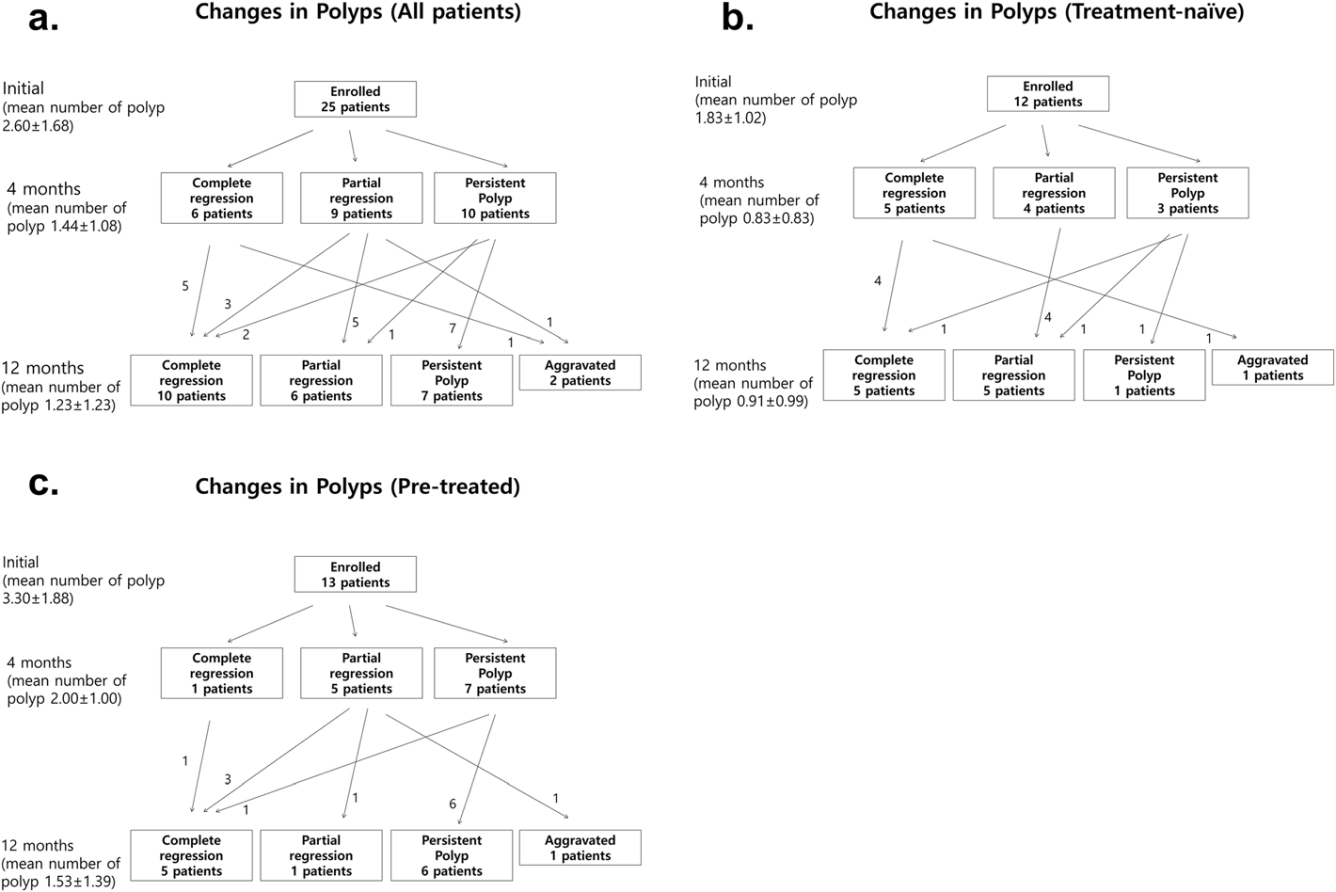
Flowcharts of polyp regression assessed with indocyanine green angiography. **a** In the all patients **b** In the treatment-naïve group **c** In the pre-treated group

Out of the 25 patients, complete polyp regression was observed in six patients (24%) at the four-month follow-up, and in ten patients (40%) at 12-month follow-up (Fig. 3).

**Fig. 4:**
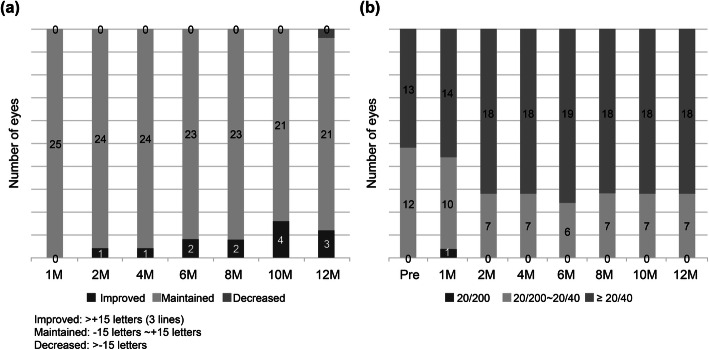
**a** Proportion of patients with improved, maintained, or decreased visual acuity. **b** Distribution of visual acuity

At 12 months, three patients (12%) showed a visual acuity improvement of 15 letters or more, and the number of patients with more than 20/40 visual acuity increased from 13 patients at baseline to 18 patients at 12 months (Fig. 4).

**Fig. 5:**
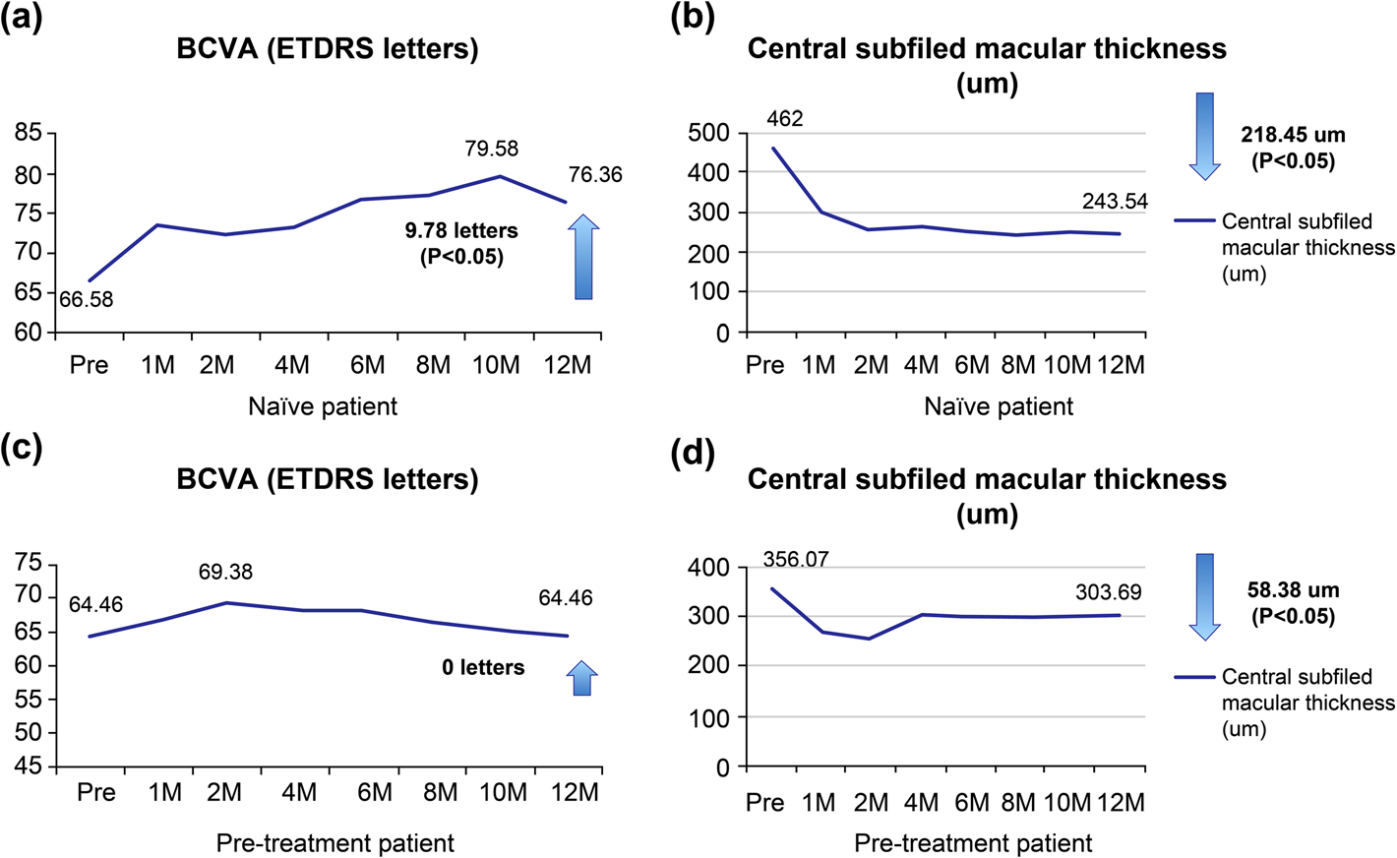
Mean change in the best-corrected visual acuity and central subfield macular thickness. **a** Mean change in the best-corrected visual acuity and **b** central subfield macular thickness of the patients in the treatment-naïve group. **c** Mean change in the best-corrected visual acuity and central subfield macular thickness of the patients in the pre-treated group. **d** central subfield macular thickness of the patients in the pre-treated group

The treatment-naïve group showed a visual acuity of improvement of 9.78 letters, from 66.58±14.79 at baseline to 76.36±6.59 at 12 months (*p*-value=0.005), and a decrease of 218.45 um in central macular thickness, from 462±160 um at baseline to 243.55±162 um at 12 months (*p*-value< 0.001). The pre-treated group also showed a decrease in central macular thickness, from 356.08±145 um at baseline to 303.69±139 um at 12 months; however, the change was not statistically significant (*p*-value=0.23). There was no change in the mean baseline visual acuity of the pre-treated group (64.46±16.52 letters) at 12 months. There was no significant difference in the complete polyp regression rates and complete subretinal fluid resolution rates of both the pre-treated and the treatment-naïve groups (Fig. 5 and Table 2). No serious systemic or ocular side effects were reported during the course of the present study.

**Table 2:** Comparison of patients in the treatment-naïve and pre-treated groups who were treated with a fixed-dosing intravitreal aflibercept regimen

	Treatment-naïve case (*n*=12)			Pre-treated (*n*=13)			*P*-value (Treatment-naïve vs. Pre-treated)
	Baseline	12 months	Δ and *P*-value	Baseline	12 months	Δ and *P*-value	
BCVA at 12 months from baseline (ETDRS letters)	66.58±14.79	76.36±6.59	Δ =9.78 letters *p*= 0.005^a^	64.46±16.52	64.46±14.61	Δ = 0 letters *p*= 1.000^a^	0.004^b^
Retinal thickness at 12 months from baseline (um)	462±160	243.55±162	Δ = 218.45 *p*= 0.000^a^	356.08±145	303.69±139	Δ = 52.39 *p*= 0.23^a^	0.009^b^
Number and Proportion of patients with complete polyp regression at month 12 n (%)		5 (41.6%)			5 (38.4%)		0.87^c^
Number and Proportion of patients with dry retina at month 12 n (%)		8 (67%)			7 (53.8%)		0.51^c^

^a^ Paired T-test ^b^ T-test ^c^ Chi-squared test)

## Discussion

In this study, the one-year outcomes of fixed-dosing aflibercept therapy for PCV patients were assessed. Improved visual acuity, which represents the functional parameters of PCV, and improved persistent subretinal fluid and polypoidal vessel signs, which represent the anatomical parameters of PCV, were observed after the treatment.

Some recent studies have evaluated the outcomes anti-VEGF therapies for the treatment of PCV patients. In the EVEREST II study, ranibizumab monotherapy was administered to PCV patients for 1 year and a 33.8% complete polypoidal lesion regression rate and a 5.1-letter improvement in visual acuity were recorded; these outcomes are inferior to those of the patients treated with ranibizumab-PDT combination therapy in the same study (69.7% complete polypoidal lesion regression rate and 8.3-letter improvement in visual acuity). The outcome of the EVEREST II study became the rationale for considering the combination with PDT as the first-line therapy for PCV [14]. However, treatment with aflibercept yielded a higher complete polypoidal lesion regression rate. In the VAULT study published in 2017, a 9.1-letter improvement in visual acuity and a 66.7% complete polypoidal lesion regression rate were recorded after 1 year of aflibercept monotherapy. These outcomes are better than those of the PDT combination group in the EVEREST II study are. The outcome of the VAULT study became the rationale for adopting aflibercept monotherapy for treating PCV [8]. In 2018, the PLANET study, which was a double-masked, sham-controlled phase, randomized clinical trial, was published; in this study, a 10.7-letter improvement in visual acuity and a 38.9% complete polypoidal lesion regression rate were recorded after aflibercept monotherapy. These results indicate that in terms of visual acuity and anatomic parameters, there is no statistically significant difference between the efficacy of aflibercept monotherapy and that of aflibercept-plus-rescue PDT [6].

In the present study, the treatment-naïve group had a 9.78-letter visual acuity improvement and a 41.6% complete polypoidal lesion regression rate. The visual acuity improvement was similar to what was recorded in the PLANET and VAULT studies, whereas the complete polypoidal lesion regression rate was similar to that of the PLANET study but lower than that of the VAULT study. In fact, the complete polypoidal lesion regression rates that has been published for aflibercept monotherapy for PCV is extremely variable, and has been reported to be up to 75% [12]. Although this variation is not ideal, it is not surprising considering the differences in the populations and the inclusion criteria of these studies. The results of the present study were not significantly different from the results of other studies on aflibercept monotherapies for PCV.

The pre-treated group in the present study did not show any statistically significant positive outcomes in terms of visual acuity improvement or decrease in retinal thickness decrease compared to the treatment-naïve group. In previous papers, improvement in visual acuity and decreased retinal thickness were recorded after the use of aflibercept for treating patients who did not respond to conventional therapies such as ranibizumab [15, 16]; however, no statistically significant improvement results were recorded for the pre-treated group in the present study. The reason for this could be that compared with the pre-treated patients in previous studies, the pre-treated group in the present study had a longer treatment period and a higher treatment frequency, which resulted in retinal pigment epithelial atrophy and reduction in retinal pigment epithelium function. Nevertheless, the improvements in the complete polypoidal lesion regression rates and the complete subretinal fluid resolution rates were similar to those of the treatment-naïve group, indicating that aflibercept monotherapy is helpful in the improvement of the anatomical outcomes of pre-treated patients as well.

The present study had some limitations. Firstly, a control group was not included for comparison, and the study population was small. Secondly, PDT was not included as a treatment option. However, despite the limitations, the present study is significant in that it is a prospective study that highlights the usefulness of aflibercept monotherapy for PCV.

## Conclusions

In conclusion, the one-year fixed-dosing aflibercept therapy is a useful treatment with favorable anatomical and functional outcomes for treatment-naïve PCV patients. It also has favorable anatomical outcomes for pre-treated PCV patients. Larger studies with a longer follow-up period and a PDT group as a control group for comparison need to be conducted in the future to help formulate adequate PCV treatment guidelines.

## Data Availability

The datasets used and/or analyzed during the current study are available from the corresponding author on reasonable request.

## Abbreviations

AMD: Age-related macular degeneration
BCVA: Best-corrected visual acuity
CSMT: Central subfield macular thickness
ETDRS: Early Treatment Diabetic Retinopathy Study
ICGA: Indocyanine green angiography
PCV: Polypoidal choroidal vasculopathy
VEGF: Vascular endothelial growth factor
